# Supplementary material for: Risk management via contemporaneous and temporal dependence structures with applications
Source: MethodsX. 2021 Nov 19;8:101587. doi: 10.1016/j.mex.2021.101587 (PMC8720887; doi:10.1016/j.mex.2021.101587)
Supplement: Supplementary Data S1 — Supplementary Raw Research Data. This is open data under the CC BY license http://creativecommons.org/licenses/by/4.0/ [file mmc1.pdf]

Available online at www.sciencedirect.com
ScienceDirect
MethodsX 00 (2021) 1–9
MethodsX
www.elsevier.com/locate/methodsX

Risk Management via Contemporaneous and Temporal Dependence structures with Applications

Emmanuel Senyo Fianu[a,b,e], Daniel Felix Ahelegbey[c,d], Luigi Grossi[e,f]

[a] *Mainz University of Applied Sciences, Lucy-Hillebrand-Str. 2, 55128, Mainz Germany and*
[b] *R+V Lebensversicherung AG, Abraham-Lincoln-Str. 46, 65189 Wiesbaden*
[c] *Boston University, MA 02215, USA, and*
[d] *University of Pavia, Corso Str. Nuova 65, 27100 Pavia, Italy*
[e] *University of Verona, Via Cantarane 24, Verona, Italy, and*
[f] *University of Padua, Via VIII Febbraio, 2, 35122 Padova, Italy*

Abstract

This paper presents the estimation methods of the Bayesian Graphical Vector Auto-regression with and without innovations such as external regressors (BG-VARX) and Bayesian Graphical Systems Equation Modelling (BG-SEM), which are developed to examine risk network structures embedded in multivariate time series. This methodical approach allows for the analysis of various dynamics and persistence in the multivariate time series in terms of risk propagation. For instance, both the BG-SEMX and BG-VARX can reveal the within-day and across-day major risk transmitters as well as risk recipients from other univariate time series, which better explain risk contagion using complex network models. In addition, the procedures for models with and without exogenous variables have been explored, which shows that the former produce more network structures compared to the latter and therefore depict their influential role. This approach, therefore, provide a platform for future research in terms of extension of the method to encompass different types of multivariate data with additional innovations that might aid feasible analysis and the design of policy instrument and the implementation of relevant policy implications.

© 2021 Published by Elsevier Ltd.

Keywords: OR in markets, complex networks, price volatility, systemic risk, multivariate time series

The paper establishes and describe:

- Development and application of innovative network models that enhances the efficient analysis of multivariate time series data.
- Estimation of intra-day and inter-day interconnection from a daily multivariate time series data and their dynamics and persistence from contagion analysis viewpoint.

☆Mainz University of Applied Sciences, Lucy-Hillebrand-Str. 2, 55128, Mainz Germany
Email address: emmanuel.fianu@lba.hs-mainz.de; emmanuelsenyo.fianu@ruv.de ()

1. Introduction

The fundamental rationale behind network theory is that essential information, knowledge, data-driven insights and patterns about complex systems can be deduced by examining the underlying network structure. Some of these systems include the following: transportation, financial markets, power grids etc. Network theory, therefore, aids the discovery of hidden structures and provide unique understanding of the interactions between complex structures and their architectural network dynamics. The use of complex networks can therefore help to extract hidden information from various complex systems. From the viewpoint of network science, complex networks can be defined as a collection of nodes connected by edges that depicts various complex interaction among the nodes. It is worth noting that almost any large system whether natural of physical exhibit some form of interconnections. In terms of the energy market, one could think of, for instance, grid lines and their interconnections, energy prices and their linkages etc. In view of this, [1] use dynamic Granger-causality and network theory to analyze the interactions among 13 European electricity spot prices, by constructing 7651 dynamic multivariate networks, where the nodes correspond to different EU countries and the links weight the Granger-causality between the variations of the respective electricity prices. Other related works include: [2], [3], [4], [5] and many more. Furthermore, accounting for linkages in electricity market zones that are rich in intermittent renewable energy sources, [6] explore volatility transmission patterns using VAR-GARCH estimation approach given ex-ante and ex-post the inauguration of a new cable. Specifically, the SAPEI cable offers two cyclical perspectives. On the one hand, it is able to accommodate stronger volatility transmissions towards Sardinia in the off-peak periods. On the other hand, it shows no significant transmissions during peak-load periods. However, this paper proposes and exploits risk network structures inherent in the Italian energy market by estimating the within-day and across-day market connections using a multivariate time series of hourly prices. Some recent papers that focus on the volatility spillover patterns include: [7], [8], [9], [10] among many others.

In consequence, various network measures, such as network density, centrality of networks, etc. have been investigated to ascertain risk propagation in the market. Our findings are relevant for market participants such as policymakers, traders, investors and regulators to guard against sudden systemic failures, which can negatively impact on many businesses and economies because of the significant socio-economic role played by energy in the global economy. Some related recent papers on systemic risk include the following: [11, 12, 13, 14, 15], who propose several econometric measures of connectedness based on principal components analysis and Granger-causality networks. According to the authors, systemic risk is inherent in financial systems and groups of interconnected institutions with business relationships, so the risk of illiquidity, insolvency and losses can quickly propagate during periods of financial distress[1].

This paper, therefore, contributes to various strands of methodological literature. First, we develop and employ an innovative Bayesian graphical network models that enhances an improved analysis of market interconnections using vector autoregression and system equation modeling with external regressors; BGVARX and BG-SEMX respectively. As a result, we provide both the within-day and across-day daily time series interconnection analysis, thereby exploring various risk network structures. On the other hand, we study the persistence of the interconnectedness in multivariate time series data. Finally, we examine the various risk network structures to identify and quantify individual univariate time series that play dominant role as well as those that are vulnerable to the spread of risk, for instance, among the multivariate time series data, thereby providing a unique understanding of risk management practices based on a multivariate time series data. Overall, modeling framework, details a methodology that is effective in designing policy and making actionable data-driven decision and thus applicable to risk management practices in various sector of the economy such as the financial markets, commodity markets, insurance markets etc.

[1]See [16] for a review of the state of the art for statistical inference and the application of network analysis to financial time series.

2. Method details

This section provides an overview of the model and estimation procedure adopted in this paper to analyze interdependencies in multivariate financial time series data. These interdependencies can be decoupled and broken down into two underlying network structure-inferred typologies: an intra-day (same day) network, in which the dependence occurs on the same day; and an inter-day (day-to-day) network, in which the dependence occurs with a time-lag. The intra-day and inter-day dependencies from multivariate time series is modeled using a simultaneous equation (SEM) and a vector autoregressive model (VAR). However, the aforementioned models also provide the possibility to incorporate exogenous variables; see [15] for further details. The subsequent sections discuss the modeling framework.

3. Bayesian Graphical Network Model Estimation

In this section, we present the Bayesian graphical framework of multivariate analysis. The procedure for Bayesian Graphical Vector Autoregression and Bayesian Graphical Structural Equation modeling framework constitute a special case of the equation (1). This implies that both methods can be represented by a system of equations framework given by:

$$Y_t = BX_t + \varepsilon_t, \qquad \varepsilon_t \sim \mathcal{N}(0, \Sigma_\varepsilon) \tag{1}$$

where $B = (B_{y|y}, B_{y|z})$, $X_t = (Y_t', Z_t')'$ and $\Sigma_\varepsilon = \Sigma_u$ in the case of the intra-day model; and $B = (A_{1,y|y}, \ldots, A_{p,y|y}, A_{y|z})$, $X_t = (Y_{t-1}', \ldots, Y_{t-p}', Z_t')'$ and $\Sigma_\varepsilon = \Sigma_v$ in the case of the inter-day model. The objective of the BG-SEMX is to estimate $(B_{y|y}, B_{y|z}, \Sigma_u)$, while that of BG-VARX is to estimate $(p, A_{y|y}, A_{y|z}, \Sigma_v)$ using the available data.

3.0.1. Network Models

Complex network analysis can be recognized as a tool for mining technological, social and financial data among many others. The multiplexity of different network structures, if accurately inferred and elicited can help provide unique understanding and insights into various naturally existing and man-made networks. As such, the introduction of networks in a typical multivariate multiple regressions model helps to interpret the relationships in the model. In the formalization of this representation, equations (1) can be specified from the viewpoint of networks by assigning to each coefficient B_{ij} a latent variable including indicator $G_{ij} \in \{0, 1\}$, such that for $i, j = 1, \ldots, n$, we have the following:

$$B_{ij} = \begin{cases} 0 & \text{if} \quad G_{ij} = 0 \quad \Longrightarrow \quad Y_j \not\to Y_i \\ b_{ij} \in \mathbb{R} & \text{if} \quad G_{ij} = 1 \quad \Longrightarrow \quad Y_j \to Y_i \end{cases} \tag{2}$$

where $Y_j \not\to Y_i$ means that Y_j does not influence Y_i. Modeling from the perspective of (1) and (2), a network model is specified by the parameters $(G, B, \Sigma_\varepsilon)$, where G is related to the latent network structure (often referred to as the adjacency matrix of a network graph). B denotes the coefficients, and Σ_ε is the residual covariance matrix.

3.0.2. Prior Specification

The joint estimation of the BG-SEMX and BG-VARX or the simplified regression model in (1) is a challenging problem and a computationally expensive. To this extent, the Bayesian formulation with prior specification and posterior approximation for the inference of the model parameters are carried out by specifying the prior distributions as follows:

$$[B_{ij}|G_{ij} = 1] \sim \mathcal{N}(0, \eta), \qquad G_{ij} \sim \text{Ber}(\pi_{ij}), \qquad \Sigma_\varepsilon^2 \sim \mathcal{W}(\delta, \Lambda_0)$$

where η, π_{ij}, δ, and Λ_0 are hyper-parameters. The specification for B_{ij} conditioned on G_{ij} follows a normal distribution with zero mean and variance η. Therefore, the relevant explanatory variables with significant information to predict a response variable are associated with coefficients different

from zero and the rest (representing not-relevant variables) are restricted to zero. We consider G_{ij} as Bernoulli distributed with π_{ij} as the prior probability. Closely related to our specification for B and G, is the stochastic search variables selection [SSVS, 17] that assumes an indicator matrix underlying B and employs the spike and slab prior on the elements in B [see also 18]. The SSVS and the Bayesian graphical VAR [BGVAR, 19] have proven efficient in selecting relevant variables in over-parameterized VAR models. The difference between the two methods is that the estimated SSVS coefficient matrix often consists of elements with values significantly different from zero, whereas the rest concentrate around zero but are not ignored. Parsimony is, therefore, not guaranteed.

Finally, we assume Σ_ε^{-1} is Wishart distributed with prior expectation $\frac{1}{\delta}\Lambda_0$ and $\delta > n$ as the degrees of freedom parameter.

3.0.3. Posterior Approximation

Let Y_t be the vector of log volatilities of the zones and X_t the vector of explanatory variables at time t. In addition, let $Y = (Y_1, \dots, Y_T)$ and $X = (X_1, \dots, X_T)$ denote a collection of Y_t and X_t over a fixed window of length T. Following the Bayesian framework of [20], the structural parameters can be integrated out analytically to obtain a marginal likelihood function over the graphs. This allows for the application of an efficient Gibbs sampling algorithm to sample the graph structure and the model parameters in blocks. In order to approximate the graph and parameters posterior distribution, we consider a collapsed Gibbs sampler that proceeds as follows:

1. Sample via a Metropolis-within-Gibbs $[G|Y, X]$ (see 3.1 for an overview)
2. Sample from $[B, \Sigma_\varepsilon|Y, X]$ by iterating the following steps:
   (a) Sample $[B_{i,\pi_i}|Y, X, \hat{G}, \Sigma_\varepsilon] \sim \mathcal{N}(\hat{B}_{i,\pi_i},\ Q_{\pi_i})$ where

$$\hat{B}_{i,\pi_i} = \sigma_{\varepsilon,i}^{-2} Q_{\pi_i} X_{\pi_i}' Y_i, \qquad Q_{\pi_i} = (\eta^{-1} I_{d_x} + \sigma_{\varepsilon,i}^{-2} X_{\pi_i}' X_{\pi_i})^{-1} \tag{3}$$

   where $X_{\pi_i} \in X$ is the set of predictors of Y_i that corresponds to $(\hat{G}_{y_i,x_\pi} = 1)$, $\sigma_{\varepsilon,i}^2$ is the i-th diagonal element of $\hat{\Sigma}_\varepsilon$, and d_x is the number of covariates in X_{π_i}.
   (b) Sample $[\Sigma_\varepsilon^{-1}|Y, X, \hat{G}, B] \sim \mathcal{W}(\delta + N,\ \Lambda_T)$ where

$$\Lambda_T = \Lambda_0 + (Y - X\hat{B}')'(Y - X\hat{B}') \tag{4}$$

For further details concerning the network sampling algorithm and convergence diagnostics, refer to 3.1 for an overview.

3.0.4. Lasso Regularization Method

We apply the Lasso regularization method by [21] as a benchmark model for our empirical analysis. This approach estimates the coefficients of (1) by solving:

$$\hat{B}_{ij} = \min_{B_{ij}} \left\{ \sum_{t=1}^{T} \left(Y_{i,t} - \sum_{j=1}^{q} B_{ij} X_{j,t} \right)^2 + \lambda \sum_{j=1}^{q} |B_{ij}| \right\} \tag{5}$$

where T is the number of observations, and q the number of covariates, λ is the penalty term, such that large values of λ shrinks a large number of the coefficients towards zero. In the empirical analysis, we select the regularization parameter using ten-fold cross-validation on a grid of λ values for the penalized logistic regression problem. We select the regularization parameter that corresponds to one standard error from the minimum mean square cross-validated errors, i.e., $\lambda.1se$.

3.1. Network Sampling Algorithms

The Bayesian inference of a network graph underlying a system of linear equations is made feasible by integrating out other parameters analytically to obtain a marginal likelihood function

over graphs [see 20, 14]. Let $V_y = (y_i, \dots, y_n)$ be the vector of indices of response variables, and $V_x = (x_1, \dots, x_q)$ the indices of the predictor variables in X. The network relationship from $x_\psi \in V_x$ to $y_i \in V_y$ can be represented by $(G_{y_i,x_\psi} = 1)$. Following [20], the closed-form expression of the local marginal likelihood is given by

$$P(Y|G_{y_i,x_\psi}) = \frac{\pi^{-\frac{1}{2}N}\ \nu_0^{\frac{1}{2}\nu_0}}{\nu_n^{\frac{1}{2}\nu_n}} \frac{\Gamma\left(\frac{\nu_0+N-n_r}{2}\right)}{\Gamma\left(\frac{\nu_0-n_r}{2}\right)} \left(\frac{|X'_\psi X_\psi + \nu_0 I_{n_v}|}{|R'_i R_i + \nu_0 I_{n_r}|}\right)^{\frac{1}{2}\nu_n} \tag{6}$$

where $\Gamma(\cdot)$ is the gamma function, $R_i = (Y_i, X_\psi)$, I_d is a d-dimensional identity matrix, n_ψ is the number of covariates in X_ψ, $n_r = n_\psi + 1$. In addition, $\nu_0 > n_r$ is a degree of freedom hyper-parameter of the prior precision matrix of (Y, X), and $\nu_n = \nu_0 + N$. Equation (6) shows that only the ratio of the posterior sum of squares depend on the data. Thus, we reduce computational time by pre-computing the part of (6) that is independent of the data, for different values of $n_r \in [1, m]$ and for fixed $\nu_0 = m + 2$ and N. We also pre-compute the posterior of the full sum of squares matrix and extract the sub-matrices that relates to $\{X_\psi\}$ and $\{(Y_i, X_\psi)\}$. For computational details of the score function [see 14].

The algorithm presented for sampling G is a Metropolis-within-Gibbs sampler with random walk proposal distribution. In the Monte Carlo Markov chain (MCMC) search algorithms, the space exploration crucially depends on the choice of the starting point of the MCMC chain. Usually, a set of burn-in iterations is conducted to obtain a good starting point. In this application, we adopt an initialization scheme that provides a good starting point for the MCMC algorithm.

3.2. Sampling SEMX Network

Let $X_t = (Y'_t, Z'_t)'$ be the vector of possible explanation variables for the SEMX model.

1. Initialization: Set $G^{(0)}$ as $(n \times n)$ null matrix.
   Set $V_y = (y_i, \dots, y_n)$ - vector of indices of response variables in Y
   Set $V_x = (x_1, \dots, x_q)$ - vector of indices of predictor variables in X.
   Note that $V_y \subseteq V_x$
2. Iterate $h = 1, \dots, H$ by performing a local network update
   Pick at random $y_i \in V_y$ and set $G^{(*)}_{y_i} = G^{(h-1)}_{y_i}$ and $V_{x_i} = V_x \backslash \{y_i\}$
   (a) Randomly draw a candidate explanatory variable $x_k \sim V_{x_i}$
   (b) **If** $G^{(h-1)}_{y_i,x_k} = 0$ and $G^{(h-1)}_{x_k,y_i} = 1$ **then**
   Consider e reverse move: $G^{(*)}_{y_i,x_k} = 1$ and $G^{(*)}_{x_k,y_i} = 0$
   else Add/remove edge between x_k and y_i: $G^{(*)}_{y_i,x_k} = 1 - G^{(h-1)}_{y_i,x_k}$
   (c) **If** G^* is acyclic **then**
   (i) Compute $\log P(Y|G^{(*)} = \sum_{i=1}^n \log P(Y|G^{(*)}_i)$
   (ii) Compute $\phi_i = \exp\ [\ \log P(Y|G^{(*)}) - \log P(Y|G^{(h-1)})\ ]$
   (iii) Draw $u \sim \mathcal{U}(0, 1)$.
   If $u < \min\{1, \phi\}$ **then**
   Set $G^{(h)} = G^{(*)}$
   else Set $G^{(h)} = G^{(h-1)}$
   (d) **else if** G^* is not acyclic
   Set $G^{(h)} = G^{(h-1)}$

3.3. Sampling VARX Network

Let $X_t = (Y'_{t-1}, \dots, Y'_{t-p}, Z'_t)'$ be the vector of possible explanation variables for the VARX model. Assume X_t is a q-dimensional vector. Following standard application, we determine the lag length p by minimizing the BIC:

$$BIC(p) \ = \ \log|\hat{\Sigma}_{y|x}(p)| + n^2 p \frac{\log M}{M}, \qquad \underline{p} \le p \le \bar{p} \tag{7}$$

where $\hat{\Sigma}_{y|x}(p) = \Sigma_{yy} - \Sigma_{yx}\Sigma_{xx}^{-1}\Sigma_{yx}'$, Σ_{yx} is the covariance between Y and X, $M = N - p$ is the number of observations, and $|\hat{\Sigma}_{y|x}(p)|$ is the determinant of $\hat{\Sigma}_{y|x}(p)$. Given some lag length $\hat{p}$, we proceed with the VAR network sampling as follows:

1. Initialization: Set $G_{|\hat{p}}^{(1)}$ as $(n \times q)$ null matrix
   Set $V_x = (x_1, \ldots, x_q)$ - vector of indices of predictor variables in X
   Set $V_y = (y_i, \ldots, y_n)$ - vector of indices of response variables in Y
   For each $y_i \in V_y$, compute a reference score: $Pr(Y|G_{y_i|\hat{p}}^{(1)})$
   (a) For each $x_j \in V_x$, compute the marginal score: $Pr(Y|G_{y_i,x_j}^{(1)})$
   (b) Compute $BF = \log Pr(Y|G_{y_i,x_j|\hat{p}}^{(1)}) - \log Pr(Y|G_{y_i|\hat{p}}^{(1)})$
   (c) **If** $BF > 0$ **then** set $G_{y_i,x_j|\hat{p}}^{(1)} = 1$ **else** set $G_{y_i,x_j|\hat{p}}^{(1)} = 0$
2. Iterate in $h = 2, \ldots, H$ by performing a local network update
   For each $y_i \in V_y$, set $G_{y_i|\hat{p}}^{(*)} = G_{y_i|\hat{p}}^{(h-1)}$
   (a) Randomly draw a candidate explanatory variable $x_k \sim V_x$
   (b) Add/remove edge between x_k and y_i: $G_{y_i,x_k|\hat{p}}^{(*)} = 1 - G_{y_i,x_k|\hat{p}}^{(h-1)}$
   (c) Compute $\phi = \exp\ [\ \log P(Y|G_{y_i|\hat{p}}^{(*)}) - \log P(Y|G_{y_i|\hat{p}}^{(h-1)})\ ]$ and draw $u \sim \mathcal{U}(0, 1)$.
   (d) **If** $u < \min\{1, \phi\}$ **then** set $G_{y_i|\hat{p}}^{(h)} = G_{y_i|\hat{p}}^{(*)}$ **else** set $G_{y_i|\hat{p}}^{(h)} = G_{y_i|\hat{p}}^{(h-1)}$

3.4. Graphical abstractions of modeling framework

This section presents the graphical abstract of our modelling framework. In effect, it summarizes some of the stages of our procedures and thus provide a unique understanding for readers with diverse interest. As earlier on mentioned, the network structures are deduced from the adjacency matrix. In our approach the sub-periods are selected based on the evolution of the network density plots which depict unique features of peaks and troughs. Furthermore, the network structures are deduced based on these sub-periods for detailed analysis. A graphical display of these procedures are demonstrated below.

Intra-day Network Analysis

$$B_{y|y} = \begin{pmatrix} 0 & 0 & 0 & 0 & 0 \\ \beta_{21} & 0 & 0 & 0 & 0 \\ \beta_{31} & 0 & 0 & 0 & 0 \\ 0 & \beta_{42} & 0 & 0 & \beta_{45} \\ \beta_{51} & 0 & \beta_{53} & 0 & 0 \end{pmatrix} \Longrightarrow$$

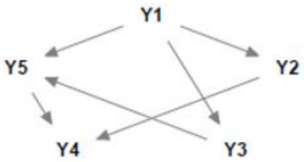

Coefficient matrix and associated network structure

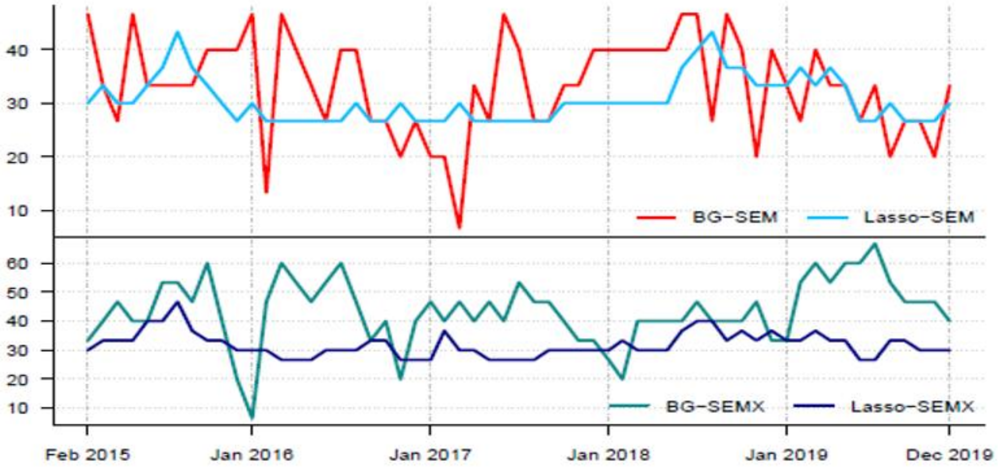

Yearly rolling series of network density from the BG-SEM & SEMX and Lasso-SEM & SEMX.

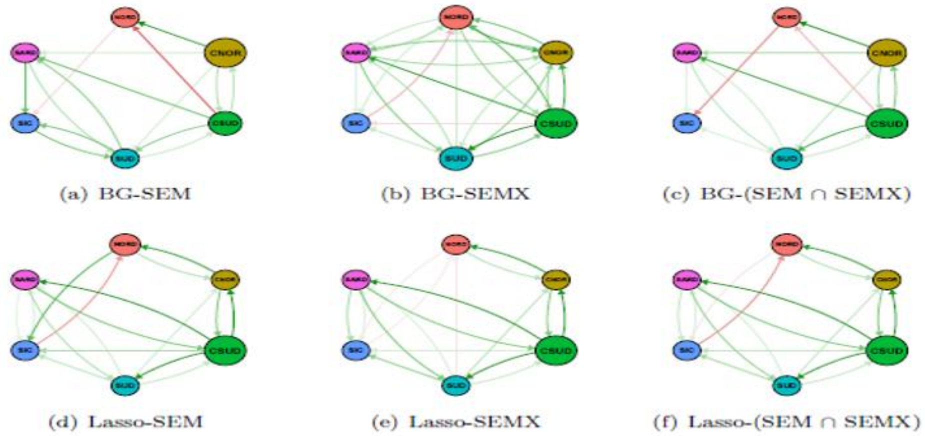

Intra-day Networks 2015–2017. Dark (light) green-links indicate strong (mild) positive impacts, and dark (light) red-links indicate strong (mild) negative effects. The node size is based on hub scores.

4. Conclusion

In this paper, methodologies based on network theory and econometrics have been presented. Specifically, we have developed and proposed the BG-VARX and BG-SEMX, which are able to account for and accommodate both the intra-day and inter-day analysis respectively. In the proposed model, multivariate time series data has been utilized to study complex dynamic network structures embedded in the data. The analyis has been achieved using, Microsoft Excel, MATLAB and R software. MATLAB recorded a maximum computational speed of BG-SEM at 9.63 secs; and the of BG-SEMX is 12.07 secs; BG-VAR is 2.67 secs; and BG-VARX is 2.80 secs.

On the one hand, for the Lasso method, we observed the following computational speed using MATLAB, that is, Lasso-SEM 2.15, Lasso-SEMX 3.38, Lasso-VAR 3.89, and for Lasso-VARX a computation speed of 6.06. These differences in the computational speed for both the BG-VAR(X) and BG-SEM(X) accounts for the constraints used in the Bayesian graphical method. On the other hand, the Lasso-SEMX and Lasso-VARX show a relatively higher computational time because of the additional covariates (or exogenous variables) and the self-looping nature of the Lasso method respectively. In this light, both intra-day and inter-day dependencies structures inherent in the data have been examined. For each case, the overall time series have been divided into sub-periods to accommodate short-term variability due to the nature of electricity time series data. The methodologies help to identify major risk transmitters and risk receivers based on the within-day and across-day dependencies respectively, which is relevant for market participants to effectively and efficiently changing trends in the markets with various underlying scenarios.

References

[1] G. Castagneto-Gissey, M. Chavez, F. D. V. Fallani, Dynamic granger-causal networks of electricity spot prices: A novel approach to market integration, Energy Economics 44 (2014) 422–432.
[2] G.-J. Wang, H.-B. Si, Y.-Y. Chen, C. Xie, J. Chevallier, Time domain and frequency domain granger causality networks: Application to chinaâ€™s financial institutions, Finance Research Letters 39 (2021) 101662.
[3] D. Wang, W.-Q. Huang, Forecasting macroeconomy using granger-causality network connectedness, Applied Economics Letters 28 (16) (2021) 1363–1370.
[4] L. Margaritella, M. Friedrich, S. Smeekes, High-dimensional granger causality for climatic attribution, in: EGU General Assembly Conference Abstracts, 2021, pp. EGU21–8020.
[5] E. Siggiridou, C. Koutlis, A. Tsimpiris, D. Kugiumtzis, Evaluation of granger causality measures for constructing networks from multivariate time series, Entropy 21 (11) (2019) 1080.
[6] A. Sapio, N. Spagnolo, Volatility import effects of a new cable linking Sardinia with mainland Italy, in conference proceedings; Energy Finance conference.
[7] L. H. Vo, T.-H. Le, Eatery, energy, environment and economic system, 1970–2017: Understanding volatility spillover patterns in a global sample, Energy Economics (2021) 105391.
[8] S. Farid, G. M. Kayani, M. A. Naeem, S. J. H. Shahzad, Intraday volatility transmission among precious metals, energy and stocks during the covid-19 pandemic, Resources Policy 72 (2021) 102101.
[9] T. Wu, X. Gao, S. An, S. Liu, Time-varying pattern causality inference in global stock markets, International Review of Financial Analysis (2021) 101806.
[10] N. T. Hung, X. V. Vo, Directional spillover effects and time-frequency nexus between oil, gold and stock markets: Evidence from pre and during covid-19 outbreak, International Review of Financial Analysis 76 (2021) 101730.
[11] M. Billio, M. Getmansky, A. W. Lo, L. Pelizzon, Econometric Measures of Connectedness and Systemic Risk in the Finance and Insurance Sectors, Journal of Financial Economics 104 (3) (2012) 535 – 559.
[12] F. Diebold, K. Yilmaz, On the Network Topology of Variance Decompositions: Measuring the Connectedness of Financial Firms, Journal of Econometrics 182 (1) (2014) 119–134.
[13] D. F. Ahelegbey, P. Giudici, Bayesian Selection of Systemic Risk Networks, Advances in Econometrics: Bayesian Model Comparison 34 (2014) 117–153.
[14] D. F. Ahelegbey, M. Billio, R. Casarin, Bayesian Graphical Models for Structural Vector Autoregressive Processes, Journal of Applied Econometrics 31 (2) (2016) 357–386.
[15] E. S. Fianu, D. F. Ahelegbey, L. Grossi, Modeling risk contagion in the italian zonal electricity market, European Journal of Operational Research.
[16] D. F. Ahelegbey, The Econometrics of Bayesian Graphical Models: A Review With Financial Application, Journal of Network Theory in Finance 2 (2) (2016) 1–33.
[17] E. I. George, D. Sun, S. Ni, Bayesian Stochastic Search for VAR Model Restrictions, Journal of Econometrics 142 (2008) 553–580.
[18] H. Ishwaran, J. S. Rao, Spike and slab variable selection: Frequentist and bayesian strategies, Ann. Statist. 33 (2) (2005) 730–773. doi:10.1214/009053604000001147.
URL https://doi.org/10.1214/009053604000001147

[19] D. F. Ahelegbey, M. Billio, R. Casarin, Bayesian Graphical Models for Structural Vector Autoregressive Processes, Journal of Applied Econometrics 31 (2) (2016) 357–386.
[20] D. Geiger, D. Heckerman, Parameter Priors for Directed Acyclic Graphical Models and the Characterization of Several Probability Distributions, Annals of Statistics 30 (5) (2002) 1412–1440.
[21] R. Tibshirani, Regression Shrinkage and Selection via the LASSO, Journal of the Royal Statistical Society. Series B (Methodological) 58 (1) (1996) 267–288.
